# Shivering management during therapeutic hypothermia in patients with traumatic brain injury: protocol from the Eurotherm3235 trial

**DOI:** 10.1186/1471-227X-15-S1-A7

**Published:** 2015-06-24

**Authors:** Jonathan KJ Rhodes, H Louise Sinclair, Claire G Battison, Bridget Harris, Peter JD Andrews

**Affiliations:** 1 Department of Anaesthesia, Critical Care and Pain Management, University of Edinburgh, Edinburgh, UK

## 

Effective hypothermia in critical care requires a strategy to prevent and manage shivering. Core body temperature in mammals is highly regulated [1]. Hypothermia leads to the activation of measures to counteract this, reducing heat loss through vasoconstriction and increasing heat generation through increased metabolism and shivering.

Shivering resists the reduction in core temperature but also increases physiological stresses, including an increase in oxygen demand, catecholamine release and hypertension. It also can appear distressing, may be confused with seizure activity and generally results in monitoring being more difficult.

In the Eurotherm3235 trial [2], the prevention of shivering required that patients were prepared for hypothermia appropriately, that shivering was detected early and that a plan to treat shivering was followed should it be detected. Patients were sedated with an opiate, propofol and/or midazolam because anesthetic agents have been shown to reduce the core temperature set point for shivering. Regular paracetamol was also prescribed as this reduces the hypothalamic temperature set point. The hands and feet of patients were covered with towels, which in addition to reducing the risk of thermal burns has been shown to suppress shivering [3]. During hypothermia, patients were observed closely for signs of shivering, particularly in the jaw, neck and trunk as these areas area the earliest to show signs of shivering [4].

On detection of possible shivering, seizures and inadequate sedation were excluded as causes of muscle movements. Specific interventions included active skin counter-warming with forced air convection as mean skin temperature contributes around 20% to the control of autonomic cold defenses, such as vasoconstriction and shivering, and 50% to thermal comfort [5], or the use of pethidine and the 2-agonist clonidine, each associated with significant anti-shivering effects [6].
